# Patient Entry of Information: Evaluation of User Interfaces

**DOI:** 10.2196/jmir.6.2.e13

**Published:** 2004-05-14

**Authors:** Matthew I Kim, Kevin B Johnson

**Affiliations:** ^1^Division of Endocrinology and MetabolismDivision of Health Sciences InformaticsJohns Hopkins University School of MedicineBaltimore MDUSA; ^2^Department of Biomedical InformaticsVanderbilt University School of MedicineNashville TNUSA

**Keywords:** Medical Records, Internet, User-Computer Interface

## Abstract

**Background:**

Personal health records are web-based applications that allow patients to directly enter their own data into secure repositories in order to generate accessible profiles of medical information.

**Objective:**

The authors evaluated a variety of user interfaces to determine whether different types of data entry methods employed by Personal health records may have an impact on the accuracy of patient-entered medical information.

**Methods:**

Patients with disorders requiring treatment with thyroid hormone preparations were recruited to enter data into a web-based study application. The study application presented sequences of exercises that prompted free text entry, pick list selection, or radio button selection of information related to diagnoses, prescriptions, and laboratory test results. Entered data elements were compared to information abstracted from patients' clinic notes, prescription records, and laboratory test reports.

**Results:**

Accuracy rates associated with the different data entry methods tested varied in relation to the complexity of requested information. Most of the data entry methods tested allowed for accurate entry of thyroid hormone preparation names, laboratory test names, and familiar diagnoses. Data entry methods that prompted guided abstraction of data elements from primary source documents were associated with more accurate entry of qualitative and quantitative information.

**Conclusions:**

Different types of data entry methods employed by Personal health records may have an impact on the accuracy of patient-entered medical information. Approaches that rely on guided entry of data elements abstracted from primary source documents may promote more accurate entry of information.

## Introduction

Personal health records (PHRs) are web-based applications that provide patients with secure access to self-generated profiles of medical information [1,2]. Currently available versions are being promoted as resources to help patients organize and track medical information collected over time from different sources [3].Expectations regarding the use of PHRs in practice are grounded in the notion that they may serve as secondary sources of information to help guide routine medical care, emergency medical care, self-monitoring, and disease management [4- 7].

As part of a previous study, we evaluated the functionality of a selection of PHRs by tracking the entry and display of profiles of representative clinical information [8]. Our investigation led us to conclude that the data entry methods employed by PHRs limit the range and content of patient-entered information related to diagnoses, prescriptions, laboratory test results, diagnostic study results, and immunizations. During the course of our study, we noted that most of the applications we evaluated prompted patients to enter information without any explicit guidance or direction. This led us to consider the question of whether different types of data entry methods employed by PHRs might have an impact on the accuracy of patient-entered information.

Over the course of the past decade, a number of investigators have contributed to a growing body of research centered on the development of heuristic standards and performance metrics to evaluate the usability of web sites [9- 12]. Most of the laboratory studies conducted by these researchers have focused on tracking the searching and navigation behavior of consumers interacting with commercial and institutional web sites [13- 15]. Those studies that have evaluated the use of patient-oriented health care web sites have tended to focus more on the accuracy and reliability of retrieved content than on usability [16- 19]. To date there have not been any published studies evaluating the performance of patients engaged in direct online entry of personal medical information.

We conducted a study to evaluate the performance of user interfaces that employ different types of data entry methods to collect patient-entered information. To simulate use of a PHR, we developed a web-based application incorporating sequences of data entry exercises. These exercises were designed to be completed by actual patients in real-time study sessions. To limit the scope of variables under consideration, we targeted patients with confirmed disorders requiring treatment with thyroid hormone preparations. This allowed us to focus on a defined range of diagnoses that may be distinguished on the basis of pathophysiologic mechanisms, diagnostic criteria, and goals of therapy. It also provided us with a unique opportunity to evaluate approaches to the entry of prescription information based on the visual identification of tablet shapes and colors.

## Methods

### Recruitment

To recruit subjects for this study, we sent messages to listed members of the American Foundation of Thyroid Patients, the National Graves' Disease Foundation, the Thyroid Foundation of America, and the Thyroid Cancer Survivors' Association [20- 23]. We also posted messages to the Usenet newsgroup at alt.support.thyroid [24]. These messages directed respondents to a recruitment web site listing information about PHRs, links to PHR web sites, information about the purpose of the study, and an online registration form. Registering respondents were sent a mailing that included study consent forms, release of information forms, medical provider information forms, pharmacy information forms, and task checklists. The task checklists asked respondents to request copies of recent clinic notes and laboratory test reports from medical providers. Respondents were asked to hold these documents in sealed envelopes for use during study sessions. Upon enrollment, each subject was sent a message listing the URL for the study web site along with a user name and password.

### Study Application

The application developed for this study was posted on a secure, password-protected web site. Subjects logging on to the web site were asked to complete a series of exercises directing them to enter information related to their diagnoses, current prescriptions, and recent laboratory test results. Each exercise focused on a discrete data entry task involving a specific type of data entry method. Interspersed pages of clearly worded instructions outlined the goal of each exercise.

To develop a typology of data entry methods, we systematically reviewed user interfaces implemented by web-based PHRs, health survey web sites, and web-based medication tracking applications [25- 39]. We stratified data entry methods on the basis of the approaches that were adopted and the user interface components were deployed to prompt entry or selection of medical information (Textbox 1). The user interfaces we developed for each exercise incorporated text boxes, pick lists, and radio button arrays that prompted the entry or selection of discrete data elements. Three different sequences of exercises were used throughout the course of the study. Each sequence followed a gradual progression from open-ended responses to constrained selections, staging the exposure of information to limit any bias that might influence subsequent responses.

**Data Entry Methods**

- Recollection
  - Free text entry
- Selection
  - Pick list /combo box selection
  - Radio button selection
  - Check box selection
- Exclusion
  - Dichotomous radio button selection
- Abstraction
  - Free text entry
  - Pick list/combo box selection

The first set of exercises in each sequence focused on the entry of diagnosis information. Subjects were directed to enter or select designations of specific disorders. Sequences of exercises prompted free text entry of recalled diagnoses, free text entry of providers' diagnoses abstracted from copies of recent clinic notes, and radio button selection of diagnoses from a categorized list (Figure 2A). Attempts were made to identify disorders on the basis of terms that might be used in discussions between providers and patients. In some instances, this called for the redundant listing of clinical, pathophysiologic, and pathologic terms relating to the same disorder (e.g. "primary hypothyroidism", "autoimmune thyroiditis", and "Hashimoto's thyroiditis"). In other instances, this allowed for the grouping of an array of different disorders under the heading of a single term (e.g. "thyroid cancer").

**Figure 2A:**
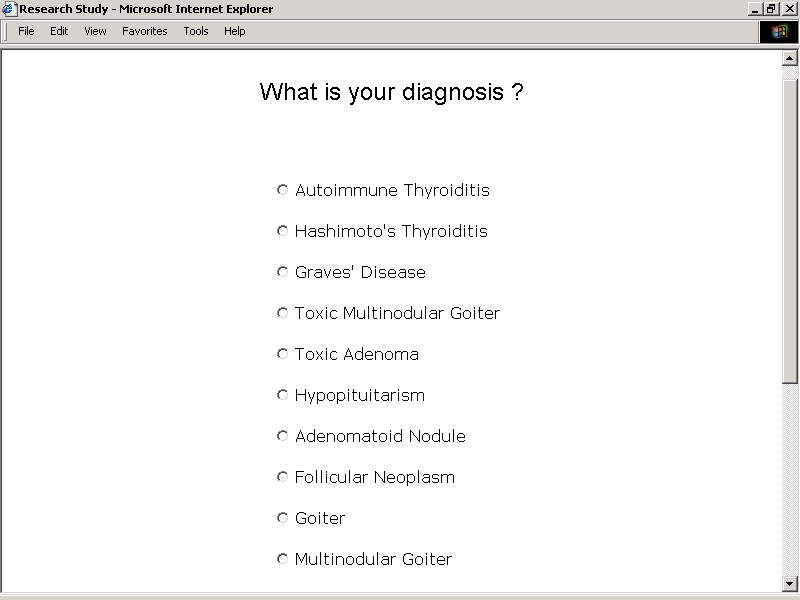
Study Application User Interfaces - Diagnoses From a Categorized List

A subset of related exercises directed subjects to identify specific goals of therapy associated with treatment with a thyroid hormone preparation. This approach sought to determine whether subjects understood distinctions between the use of thyroid hormone for replacement to correct primary deficiencies, replacement to correct secondary deficiencies, suppression to prevent growth of benign tissue, and suppression to prevent growth of malignant tissue. Understanding at this level may have a bearing on the interpretation of laboratory test results used to monitor responses to treatment [40,41]. Sequences of identification exercises prompted free text entry of recalled goals of therapy (Figure 2B), radio button selection of goals of therapy from a categorized list, and dichotomous radio button selection of answers to a series of exclusionary yes/no questions (Figure 3).

**Figure 2B:**
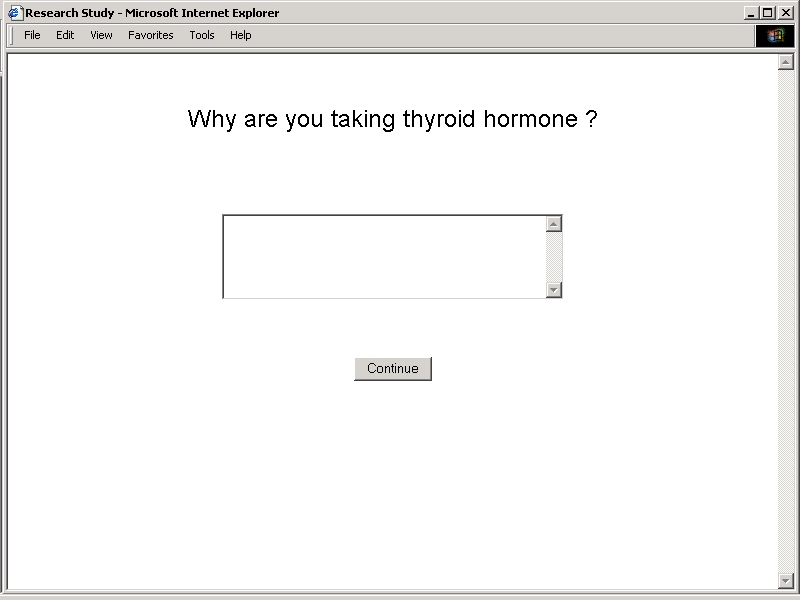
Study Application User Interfaces - Recalled Goals of Therapy

**Figure 3:**
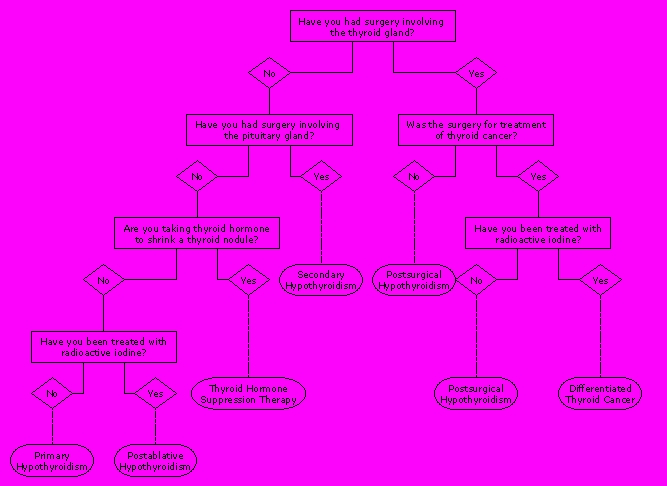
Exclusionary Questions

The second set of exercises in each sequence focused on the entry of prescription information. Subjects were directed to enter or select names of specific thyroid hormone preparations along with the strength, units, amount, and frequency of prescribed doses. A designation exercise prompted free text entry of recalled name, dose, number, and frequency information without any reference to prescription labels. A secondary designation exercise prompted radio button selection of a name from a categorized list. Visual identification exercises directed subjects to inspect their thyroid hormone tablets. This exercise took advantage of the fact that (1) three of the major brands of levothyroxine produced in the United States are manufactured as distinctively shaped tablets, and (2) levothyroxine tablets of different strengths are dyed particular colors according to a conventional scheme. As part of one exercise, subjects were prompted to select tablet shapes and imprints from an array of line drawings (Figure 2C).

**Figure 2C:**
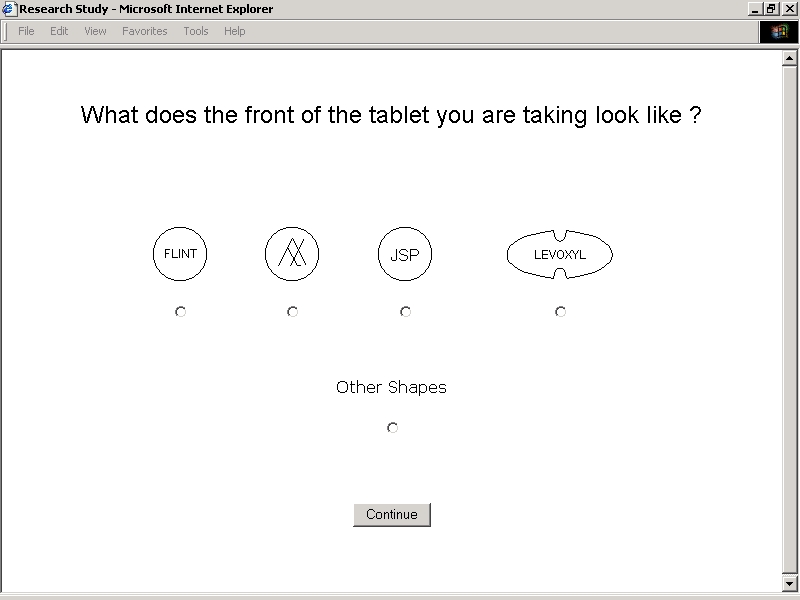
Study Application User Interfaces - Tablet Shapes and Imprints

**Figure 2D:**
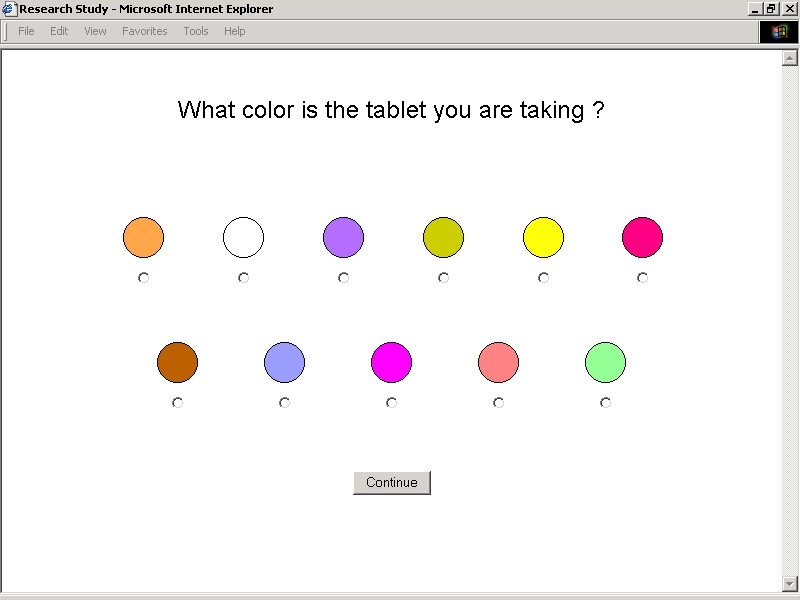
Study Application User Interfaces - Color Selection

As part of a separate exercise, subjects were prompted to select colors from an array of swatches (Figure 2D). To complete each selection and visual identification exercise, subjects were asked if each preparation was prescribed as a standard amount (one tablet) at a standard frequency (once daily). Subjects who identified nonstandard dosing regimens were prompted to select the number of tablets taken on each day of the week from an array of pick lists divided into half-tablet increments. This approach was adopted to approximate prescription instructions that are commonly issued when nonstandard doses of thyroid hormone are used to suppress the growth of benign or malignant tissue.

**Figure 2E:**
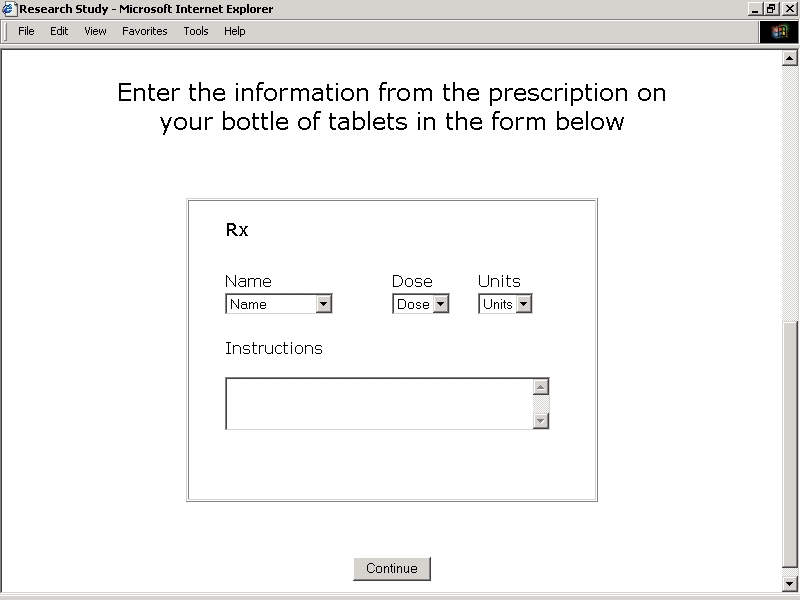
Study Application User Interfaces - Blank Prescription Using Guided Entry of Text or Guided Selection from Pick Lists

A third exercise directed subjects to review printed information appearing on their thyroid hormone prescription labels. Subjects then were prompted to enter the name, strength, units, amount, and frequency into fields similar in appearance those on a blank prescription using guided entry of text or guided selection from pick lists (Figure 2E). Highlighted samples of completed prescription labels were provided for review. Comprehensive pick lists included generic names, brand names, doses in milligrams, doses in micrograms, and amounts listed in half-tablet increments.

**Figure 2F:**
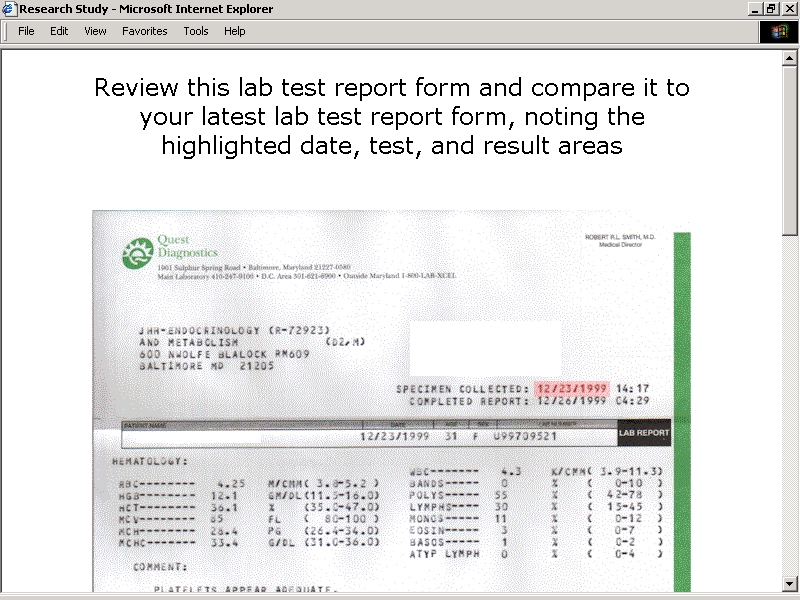
Study Application User Interfaces - Picking Out Specific Report Components

The third set of exercises in each sequence focused on the entry of laboratory test result information. Subjects were directed to enter instances of specific results and identifying information that was associated with a range of tests commonly used to monitor the treatment of thyroid disorders. A designation exercise prompted free text entry of any recalled test names and results. A secondary abstraction exercise prompted free text entry of test names, results, and dates abstracted from entries appearing in copies of recent clinic notes. Primary abstraction exercises directed subjects to review copies of test reports. An initial exercise prompted free text entry of any abstracted information deemed to be important without any specific guidance or instruction. This exercise was followed by prompted entry of abstracted information into arrays of text boxes associated with specific test names. Users were asked to enter the laboratory name and the test date along with a result, unit, upper limit of reference range, and lower limit of reference range for each test. A sample of a composite test report was provided for review, along with a glossary of synonyms and abbreviations associated with different test names. An alternate version of this exercise took advantage of the fact that a significant percentage of laboratory tests ordered in the United States are performed by two commercial laboratories. These laboratories use standard forms to report results associated with designated test names, units, and reference ranges. Subjects were directed to inspect copies of test reports to determine if they bore the logo of one of these commercial laboratories. Subjects identifying commercial test reports were directed to review scanned copies of standard forms highlighted to pick out specific report components (Figure 2F). Text boxes prompted entry of the test date along with a result for each test.

### Medical Record Analysis

Subjects' medical providers were contacted to obtain information to be used for reference purposes. Copies of signed release of information forms were faxed to provider offices along with documents requesting faxed or mailed copies of the subjects' most recent clinic notes, consultation communications, and laboratory test reports. Names of relevant disorders were abstracted from the headings of "Impression" and "Assessment" entries listed in problem-oriented clinic notes. Entries listed in consultation communications were given precedence over those listed in clinic notes in cases where there were points of disagreement. Relevant test names and results were abstracted from laboratory reports along with identifying information including laboratory names, test dates, units, and upper and lower limits of reference ranges. Designated pharmacies were contacted directly by phone to confirm recent prescription information. In each case, the last confirmed prescription issued prior to completion of the study was used as a basis for establishing a reference date, preparation, strength, amount and frequency.

### Data Analysis and Statistical Methods

Accuracy rates for the entry of different data elements were calculated by comparing entered information to confirmed reference standards. Names and designations entered as free text were checked for spelling errors. When appropriate, designations entered as free text were analyzed to determine whether they included extraneous information. Comparisons between accuracy rates associated with different user interfaces were based on Fisher's exact test calculations which were performed using STATA statistical software. Institutional Review Board approval was obtained prior to beginning this study.

## Results

Fifty-one respondents registered for the study. Fourteen registered respondents completed and returned all of the forms necessary for enrollment in the study. Eleven of the subjects who enrolled in the study successfully completed all of the exercises included in the study application. Copies of recent clinic notes and laboratory test reports were obtained from the designated medical providers who were listed for all of the subjects who completed the study. Recent prescription information was confirmed for all of the subjects who completed the study.

### Diagnosis

Eleven subjects were prompted to enter recalled diagnoses as free text (Table 1). All of these subjects entered text strings that included a correct diagnosis. Two subjects misspelled the diagnoses. Five subjects included extraneous information (e.g., a subject with a diagnosis of "papillary thyroid cancer" entered "stage IV differentiated carcinoma with marginal extension and Hurthle cell features"). Eight subjects were prompted to abstract diagnoses from copies of recent clinic notes. Seven of these subjects entered text strings that included a correct diagnosis. Four subjects misspelled the diagnoses. Four subjects included extraneous information. Nine subjects were prompted to select a diagnosis from a categorized list. Eight of these subjects selected a correct diagnosis.

**Table 1:** Diagnosis: Name

Data entry method	Recollection	Abstraction	Selection	
	- Free text entry	- From clinic notes - Free text entry	- Radio button selection	
	N = 11	N = 8	N = 9	p
Correct name	(11) 100	(7) 87.5	(8) 88.9	0.505
Correct spelling	(9) 81.8	(4) 50	(9) 100	0.047
No extraneous information	(6) 54.5	(4) 50	(9) 100	0.033
Results reported as (number) percentage				

Eleven subjects were prompted to enter recalled goals of therapy as free text (Table 2). Three of these subjects entered text strings that included a correct principal goal of therapy. Five of the remaining subjects entered a correct related goal of therapy. Eleven subjects were prompted to select a goal of therapy from a categorized list. Six of these subjects selected a correct principal goal of therapy. All of the remaining subjects selected a correct related goal of therapy. Eleven subjects were prompted to identify goals of therapy by selecting answers to a series of exclusionary yes/no questions. All of these subjects identified a correct principal goal of therapy.

**Table 2:** Diagnosis: Goal of Therapy

Data entry method	Recollection	Selection	Exclusion	
	- Free text entry	- Radio button selection	- Radio button selection	
	N = 11	N = 11	N = 11	p
Correct principal goal	(3) 27.3	(6) 54.5	(11) 100	0.001
Correct spelling	(11) 100	(11) 100	(11) 100	0.014
Related goal	(5) 62.5	(5) 100	N/A	0.196
Results reported as (number) percentage				

### Prescriptions

Nine subjects were prompted to enter recalled prescription information as free text. In each of these 12 instances, the subjects entered text strings that included a correctly spelled generic or trade name (Table 3). In eight instances these subjects entered correct strengths, in six they entered correct units, in three they entered correct frequencies of administration, and in two they entered correct amounts administered.

Nine subjects were prompted to select generic or trade names from a categorized list. Eight of these subjects selected correct preparations. Ten subjects were prompted to select tablet shapes and imprints from an array of line drawings. In each of the 14 instances these subjects selected correct preparations. Ten subjects were prompted to select colors from an array of swatches. In 8 of 14 instances these subjects selected correct preparations. All of the subjects selecting names, tablet shapes, tablet imprints, and color swatches were prompted to select amounts administered and frequencies of administration from pick lists. In 32 of 37 instances these subjects selected the correct amounts administered and frequencies of administration.

Seven subjects were prompted to enter information abstracted from prescription labels as free text. All of these subjects entered text strings that included correctly spelled names, correct amounts administered, and correct frequencies of administration. Six subjects entered correct units, while four entered correct strengths. Seven subjects were prompted to select information abstracted from prescription labels from pick lists. All of these subjects selected correct names, strengths, units, amounts administered, and frequencies of administration.

**Table 3:** Prescription

Data entry method	Recollection	Selection			Abstraction		
	- Free text entry	- Radio button Selection - Names	- Radio button Selection - Shapes	- Radio button selection - Colors	- From Prescription labels - Free text entry	- From Prescription labels - Pick list selection	
	N = 12	N = 9	N = 14	N = 14	N = 7	N = 7	p
Correct name	(12) 100	(8) 88.9	(14) 100	(14) 100	(7) 100	(7) 100	0.365
Correct spelling	(12) 100	(9) 100	(14) 100	(14) 100	(7) 100	(7) 100	-
Correct strength	(8) 66.7	(8) 88.9	(14) 100	(8) 57.1	(4) 57.1	(7) 100	0.013
Correct units	(6) 50	(9) 100	(14) 100	(14) 100	(6) 85.7	(7) 100	0.001
Correct amount	(2) 16.7	(32) 86.5			(7) 100	(7) 100	0.001
Correct frequency	(3) 25	(32) 86.5			(7) 100	(7) 100	0.001
Results reported as (number) percentage							

### Laboratory Test Results

Four subjects elected to enter recalled laboratory test information as free text (Table 4). All of these subjects entered text strings that included correctly spelled test names. One subject entered a correct result.

Nine subjects were prompted to enter laboratory test information abstracted from recent clinic notes as free text. In each of the 11 instances these subjects entered text strings that included correct test names. In one instance a subject misspelled a test name. In 10 instances these subjects entered correct results, while in eight they entered correct dates.

Eight subjects were prompted to enter laboratory test information abstracted from copies of general test reports without any guidance. In each of these11 instances the subjects entered text strings that included correct test names. In one instance a subject misspelled a test name. In nine instances these subjects entered correct results, in three they entered correct dates, in two they entered correct units, and in one instance a subject entered correct upper and lower limits of reference ranges. None of these subjects entered correct laboratory names.

**Table 4:** Laboratory Test Results

Data entry method	Recollection	Abstraction				
	- Free text entry	- From clinic notes - Free text entry	- From general reports, without guidance - Free text entry	- From general reports, with guidance - Free text entry	- From commercial forms - Free text entry	
	N = 4	N = 11	N = 11	N = 13	N = 8	p
Correct laboratory	N/A	N/A	(0) 0	(12) 92.3	(8) 100	0.001
Correct date	N/A	(8) 72.7	(3) 27.3	(10) 76.9	(6) 75	0.058
Correct test	(4) 100	(11) 100	(11) 100	(13) 100	(8) 100	-
Correct spelling	(4) 100	(10) 90.9	(10) 90.9	(13)100	(8) 100	0.735
Correct result	(1) 25	(10) 90.9	(9) 81.8	(13) 100	(8) 100	0.003
Correct units	N/A	N/A	(2) 18.2	(7) 53.8	(8) 100	0.001
Correct upper limit	N/A	N/A	(1) 9.1	(13) 100	(8) 100	0.001
Correct lower limit	N/A	N/A	(1) 9.1	(13) 100	(8) 100	0.001
Results reported as (number) percentage						

Nine subjects were prompted to enter laboratory test information abstracted from copies of general test reports with specific guidance. In each of the13 instances these subjects entered text strings that included correctly spelled test names, correct results, and correct upper and lower limits of reference ranges. In 12 instances these subjects entered correct laboratory names, in 10 they entered correct dates, and in seven they entered correct units. Six subjects elected to enter laboratory test information abstracted from copies of commercial forms with specific guidance. In each of these eight instances the subjects entered text strings that included correct results, prompting automatic selection of correctly spelled test names, units, and upper and lower limits of reference ranges. In six instances subjects entered correct dates.

## Discussion

This study demonstrated that different types of data entry methods may have an impact on the accuracy of patient-entered information. Within each defined category, accuracy rates associated with different data entry methods appeared to vary in relation to the complexity of requested information.

Free text entry of recalled or abstracted information proved to be a fairly accurate means of entering the names of specific diagnoses. This finding was somewhat reassuring in light of the fact that most of the PHRs in current use rely on free text entry of recalled information as a principal data entry method [25- 33]. It was interesting to note that subjects entering free text designations were more apt to make spelling errors in the course of entering information abstracted from clinic notes. We initially attributed these errors to illegible handwriting. Review of copies of clinic notes revealed that all but one were typewritten transcriptions of dictated entries. An alternative explanation may lie in the fact that the most of these entries included elements of medical jargon that may not be familiar to patients. This raises the question of whether diagnosis information entered as free text may need to be processed by spell-checkers that recognize acronyms and abbreviations used in clinical documentation. Subjects entering free text designations were more apt to include extraneous information that did not contribute to identification of a primary diagnosis. Most of this extraneous information focused on the assignment of etiologies or estimations of the severity of symptoms. While these modifiers did not necessarily detract from designations under consideration, their presence raised the question of whether diagnoses entered as free text may need to be parsed and sorted to isolate data elements of interest.

When entry of diagnosis information was extended to include goals of therapy, free text entry of recalled information proved to be a less accurate means of identifying principal goals of therapy. This finding was somewhat surprising in light of the fact that most of the subjects were taking prescribed thyroid hormone preparations for purposes of replacement or suppression, which are two well defined models of cause-and-effect relationships. Subjects did not fare any better in attempting to select principal goals of therapy from a categorized list of statements. The approach that focused on the selection of answers to a series of exclusionary yes/no questions proved to be the most accurate means of directing subjects to identify principal goals. This raises the question of the extent to which patients may be relied upon to directly identify their own goals of therapy. Distinction at this level may be important in situations where patients are taking agents that may be prescribed for the treatment of different conditions (e.g., diuretics, beta-blockers, systemic glucocorticoids, antiseizure medications, immunosuppressive medications). Whenever feasible, an indirect approach based on dichotomous responses to structured questions may prove to be a more reliable method of self-directed categorization.

Free text entry of recalled information was an accurate means of identifying specific names and strengths of different thyroid hormone preparations. This might have been anticipated, given the high likelihood of each subject's familiarity with this information when refilling prescriptions. For reasons that were not clear, subjects were less apt to include accurate quantitative information about units, amounts administered, and frequencies of administration in separate free text entries. This omission may have been based on the notion that this information was implicit, given the widespread use of standard dosing. It seemed less likely that this was due to lack of awareness, given that subjects following standard and nonstandard dosing regimens were able to select accurate quantitative information from pick lists. Visual identification exercises revealed that selection of tablet shapes and imprints led to more accurate identification of preparations than selection of color swatches. This discrepancy may have arisen as a result of differential browser settings, monitor settings, or variations in color perception. It should be noted that the approach based on the selection of distinctive outlines may have been successful due to the fact that all the subjects who completed this exercise were taking distinctive brand name preparations of thyroid hormone. This mode of identification may be limited in settings where the use of generic preparations that vary in shape and appearance may be more common. Direct abstraction of information from prescription labels proved to be an accurate means of entering identifying and quantitative information, irrespective of whether data elements were entered as text or selected from pick lists. Guided text entry of abstracted information might offer the advantage of greater flexibility in situations where highly variable dosing regimens may preclude generation of comprehensive pick lists (e.g., insulin regimens, immunosuppressive regi mens, adjustments of doses in chronic renal failure).

Exercises that focused on the entry of laboratory test result information suggested that the success of each approach depended in part on the source material selected for review and the degree of guidance provided in directing the abstraction of information. While subjects who engaged in free text entry of recalled information were able to identify recent tests, they were less successful in attempts to report quantitative results. Interestingly, subjects who were able to locate test results in the context of clinic notes were generally able to abstract and enter accurate qualitative and quantitative information. This exercise may have been facilitated in part by the fact that most providers documented tests of interest, results, and subsequent directives using unambiguous telegraphic styles of reporting. Approaches that rely on this mode of secondary abstraction may be confounded in situations where providers choose to document directives as annotations to laboratory test reports. Entry of a full range of qualitative, identifying, and quantitative data elements relied on directing subjects to review and abstract information from actual copies of test reports. When left to their own devices, most subjects failed to account for the source, date, units, and limits of reference ranges specified for reported results. The need for this level of detail would likely depend on the anticipated use of this information. Tracking of instances of laboratory testing might only require accurate input of source, date, and test and information. Entry of laboratory test results for purposes of disease management or self-monitoring would likely depend on accurate input of a complete range of data elements. Direct abstraction of laboratory test result information from actual copies of test reports proved to be more successful when subjects were provided with specific guidance regarding the identity, location, and format of requested data elements appearing in printed summaries. For reasons that were unclear, the only discrepancy in the accuracy of input noted was associated with the entry of unit information for requested test results. On the whole, the accuracy of guided abstraction from general format test reports appeared to match that of guided abstraction from standard commercial forms. In this case, accurate entry of information appeared to depend more on the amount of guidance provided than on the degree of constraint imposed on the range of possible entries.

The approach we adopted in designing this study had limitations. Most of the subjects we recruited were members of thyroid patient organizations and support groups. These subjects might be expected to have a certain amount of familiarity with the terminology used to describe different thyroid disorders, thyroid hormone preparations, and thyroid function tests. This may have led to overestimation of the accuracy of data entry methods. On balance, we considered this to be an acceptable risk, given some initial concerns we had about maintaining subjects' interest in participation throughout the course of the study. These concerns appeared to be borne out by the observation that a low percentage of the respondents who registered for the study actually enrolled as participants.

We chose to focus on entry of a relatively narrow range of information drawn from the domain of a particular medical subspecialty. This may have oversimplified the process of information collection by directing subjects to focus on isolated data elements. Exclusion of other diagnostic and therapeutic information may have curtailed any confusion that might have been encountered in the setting of more complex medical histories or prescription regimens.

Many of the exercises included in the study relied on the abstraction of information from documents requested directly from medical providers. While most of the subjects who were enrolled in the study were able to obtain the necessary documents with little if any difficulty, it is unclear whether this experience would be generalizable to the population at large. Given concerns about issues of liability and confidentiality, it might be reasonable to expect that patients who attempt to request documents from medical providers may encounter varying degrees of resistance. Most of the patient-oriented document organization systems in use today advocate this approach to the collection of medical information [42,43].

### Conclusions

Different data entry methods employed by PHRs appear to have an impact on the accuracy of patient-entered medical information. Strategic approaches adopted in planning the design of personal health records may need to take intended uses and purposes of entered information into account. Free text entry of recalled information may serve as an adequate means of entering simple designations of diagnoses, prescriptions, and laboratory tests. Accurate entry of more detailed qualitative and quantitative information may necessarily rely on approaches that prompt the guided entry of data elements abstracted from primary source documents. Further investigation should focus on evaluation of the accuracy of patient-directed entry of the full range of information that comprises a detailed medical history.

## Abbreviations

PHR: Personal health record
